# Therapeutic roles of platelet-rich plasma to restore female reproductive and endocrine dysfunction

**DOI:** 10.3389/fendo.2024.1374382

**Published:** 2024-04-09

**Authors:** Xiaoning Wang, Jin Li, Weiwei Lu, Fangbo Gao, Songling Zhang, Jiajia Li

**Affiliations:** ^1^Department of Blood Transfusion, The First Hospital of Jilin University, Changchun, Jilin, China; ^2^Department of Gynecologic Oncology, Gynecology and Obstetrics Centre, The First Hospital of Jilin University, Changchun, Jilin, China; ^3^The Laboratory of Cancer Precision Medicine, The First Hospital of Jilin University, Changchun, Jilin, China

**Keywords:** platelet-rich plasma, infertility, reproductive, endocrine dysfunction, female

## Abstract

Millions of women worldwide are infertile due to gynecological disorders, including premature ovarian insufficiency, polycystic ovary syndrome, Asherman syndrome, endometrial atrophy, and fallopian tube obstruction. These conditions frequently lead to infertility and have a substantial impact on the quality of life of the affected couples, primarily because of their psychological implications and high financial costs. Recently, using platelets to stimulate cell proliferation and tissue differentiation has emerged as a promising approach in regenerative medicine. Platelet-rich plasma (PRP) shows considerable potential for promoting endometrial hypertrophy and follicle development, making it a promising therapeutic option for tissue repair or replacement. This review provides an overview of the recent advancements and underlying mechanisms of PRP therapy for various female reproductive diseases and presents new therapeutic options for addressing female infertility.

## 1 Introduction

The study of reproductive endocrinology in women’s health is an enigmatic and intricate topic in the life sciences. Reproductive endocrinology encompasses disorders across the entire lifespan of a woman, including abnormalities in adolescence and sexual development, menstrual and menopausal disorders and treatments, infertility, assisted reproductive technology (ART), and recurrent abortions. Female factor infertility accounts for approximately one-third of all infertility cases (1). Diseases of the reproductive system are the primary factors contributing to female infertility. These diseases encompass ovulation dysfunction, such as premature ovarian failure (POF), polycystic ovary syndrome (PCOS), tubal infertility, endometriosis, and uterine and cervical causes, such as Asherman syndrome (AS) (1, 2). Despite the global prevalence of female infertility, recent developments in its diagnosis and treatment have been limited. Addressing female reproductive endocrine disorders is an urgent global challenge (3).

**Figure 1:**
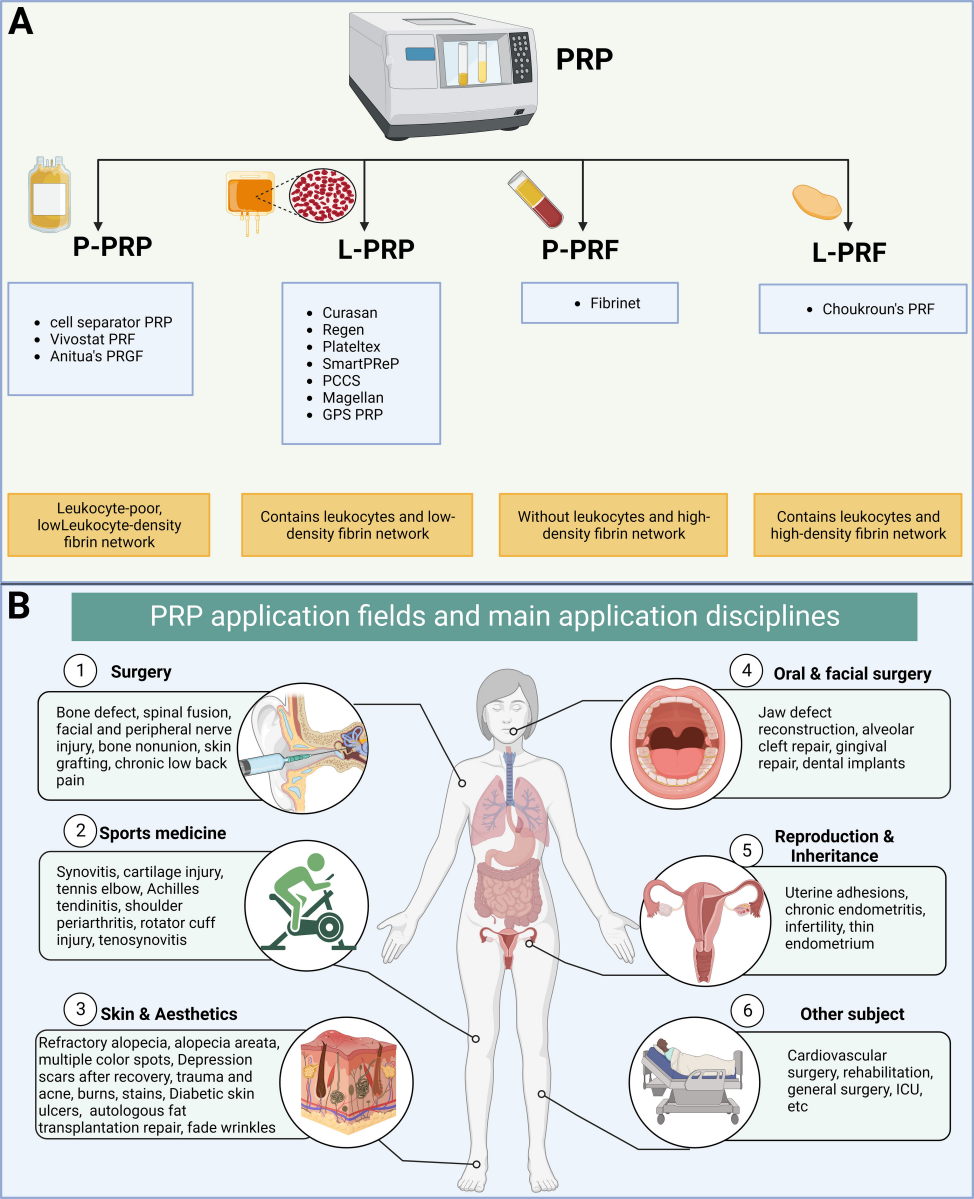
Classification and clinical application of platelet-rich plasma (PRP). **(A)** Classification of platelet concentrates based on the presence of cell content and fibrin architecture. **(B)** Fields and disciplines of PRP applications.

Platelet-rich plasma (PRP) is a substance derived from a person’s own blood that contains a high concentration of platelets, these are small blood cells that play a crucial role in clotting and wound healing, exhibiting minimal immune rejection (4–6). Platelet concentrates are categorized into four types based on white blood cell and fibrin content: pure PRP, white blood cell and PRP, pure platelet-rich fibrin, and white blood cell and platelet-rich fibrin (7). PRP has been proposed as a promising therapeutic strategy for repairing or replacing damaged tissues or organs, representing a form of regenerative medicine (4–6) (**Figure 1**). Recent clinical studies have used PRP to treat the female reproductive system and have yielded impressive results. For instance, PRP has shown the potential to promote endometrial and follicle growth, although its specific mechanism remains unclear (6, 8, 9).

This review elucidates the manifold functions of PRP in female reproductive and endocrine disorders, addressing the pathogenesis and advanced therapies for female infertility.

## 2 PRP is a promising therapeutic approach for female infertility

**Figure 2:**
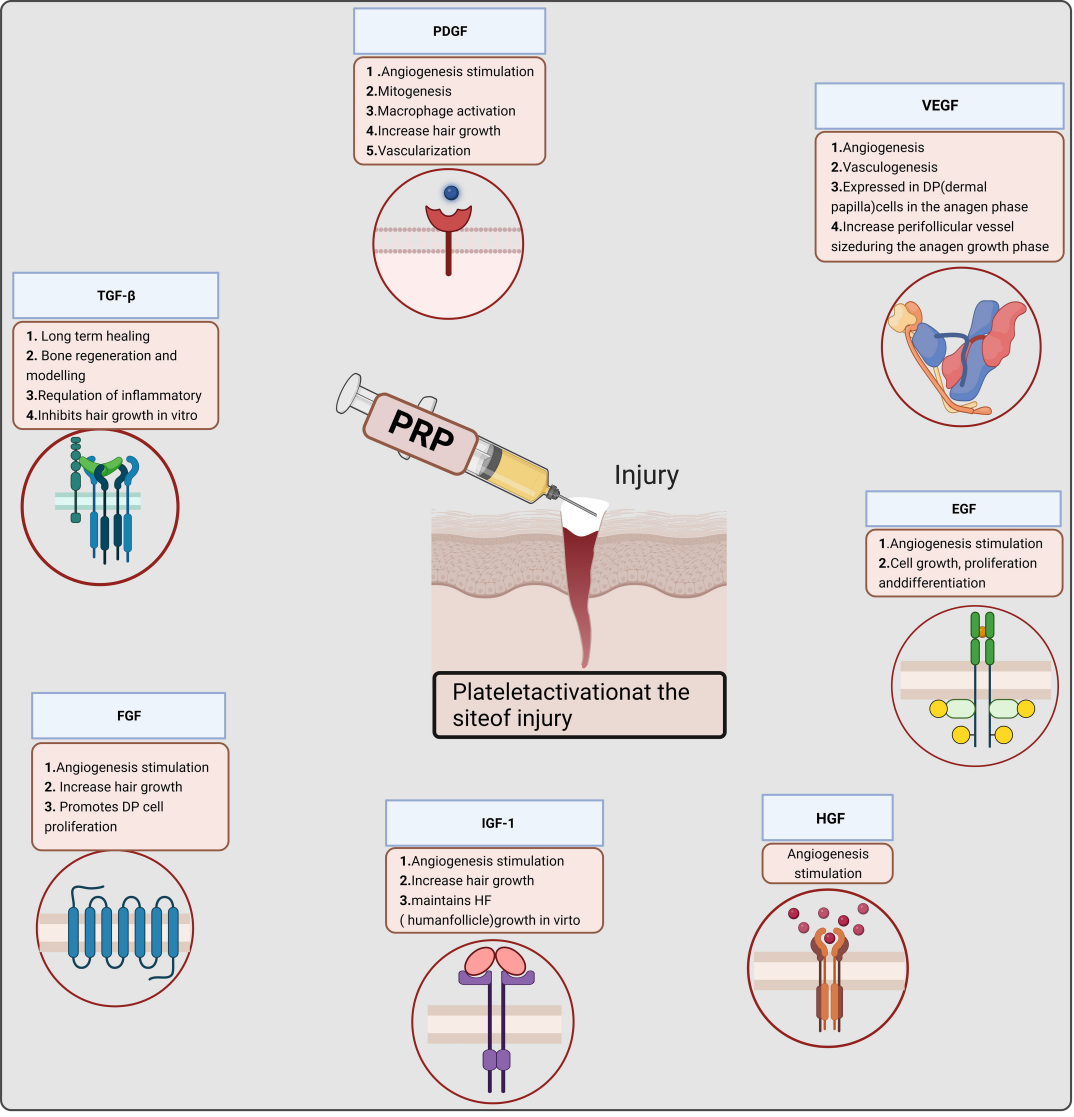
Mechanism of platelet-rich plasma (PRP) in cell proliferation. P-PRP, pure platelet-rich plasma; L-PRP, leucocyte- and platelet-rich plasma; P-PRF, pure platelet-rich fibrin; L-PRF, leucocyte- and platelet-rich fibrin.

PRP contains seven fundamental proteins: platelet-derived growth factor (PDGF), transforming growth factor beta (TGF-β), vascular endothelial growth factor (VEGF), epidermal growth factor (EGF), hepatocyte growth factor (HGF), fibroblast growth factor, and insulin-like growth factor 1 (4–6, 10). Various pathways trigger cell growth, proliferation, and differentiation (11) (**Figure 2**). For instance, PDGF activates membrane receptors on target cells, forming high-energy phosphate bonds that, in turn, activate specific cellular activities, such as mitosis, angiogenesis, and macrophage activation, produced by platelets and macrophages. TGF-β is an anti-proliferative factor in epithelial cells (12), fibroblasts, bone marrow stem cells, and preosteoblasts are TGF-β target cells. VEGF, a signal transduction protein secreted by cells, stimulates angiogenesis (13). In the local environment, PRP’s bioactive molecules play four major roles. They are involved in cell proliferation, migration, differentiation, and angiogenesis. PRP enhances angiogenesis, inhibits apoptosis, and extends cell growth (11, 14, 15) (**Figure 2**). Various factors in PRP positively affect wound healing and promote tissue regeneration and repair. PRP has been extensively utilized in multiple clinical disciplines and applied in reproductive medicine.

In the United States, approximately 13% of women of reproductive age seek infertility treatment annually. Female reproductive and endocrine disorders encompass ovulation disorders [such as PCOS, hypothalamic dysfunction, and primary ovarian insufficiency (POI)], congenital gonadal hypoplasia, tubal infertility, endometriosis, and AS (2, 3). Current treatment options, including hormone replacement therapy (HRT), induced ovulation, ovarian tissue cryopreservation transplantation, and ART, have partially alleviated symptoms, restored hormone levels, and met fertility requirements (2, 16). Despite these advancements, instances of treatment failure, deficiencies, and potential safety concerns remain, with many women having reproductive and endocrine disorders and requiring improved treatment options or strategies. Restoring normal ovarian and endometrial cycles is crucial to ensure reproductive and endocrine functions, primarily involving follicle development and endometrial hypertrophy. Researchers have explored additional methods to restore ovarian and endometrial function and treat infertility, such as PRP. PRP transplantation in patients with POI has demonstrated the potential to aid the recovery of ovarian function and enhance pregnancy success. Additionally, PRP treatment can alleviate ovarian dysfunction caused by PCOS by enhancing the ovarian antioxidant potential and folliculogenesis (17). Furthermore, PRP can enhance endometrial receptivity and increase the *in vitro* fertilization (IVF) success rate (18, 19). Moreover, PRP promotes endometrial hypertrophy and restores menstrual volume in patients with AS (20).

## 3 PRP therapy for conditions causing female infertility

**Figure 3:**
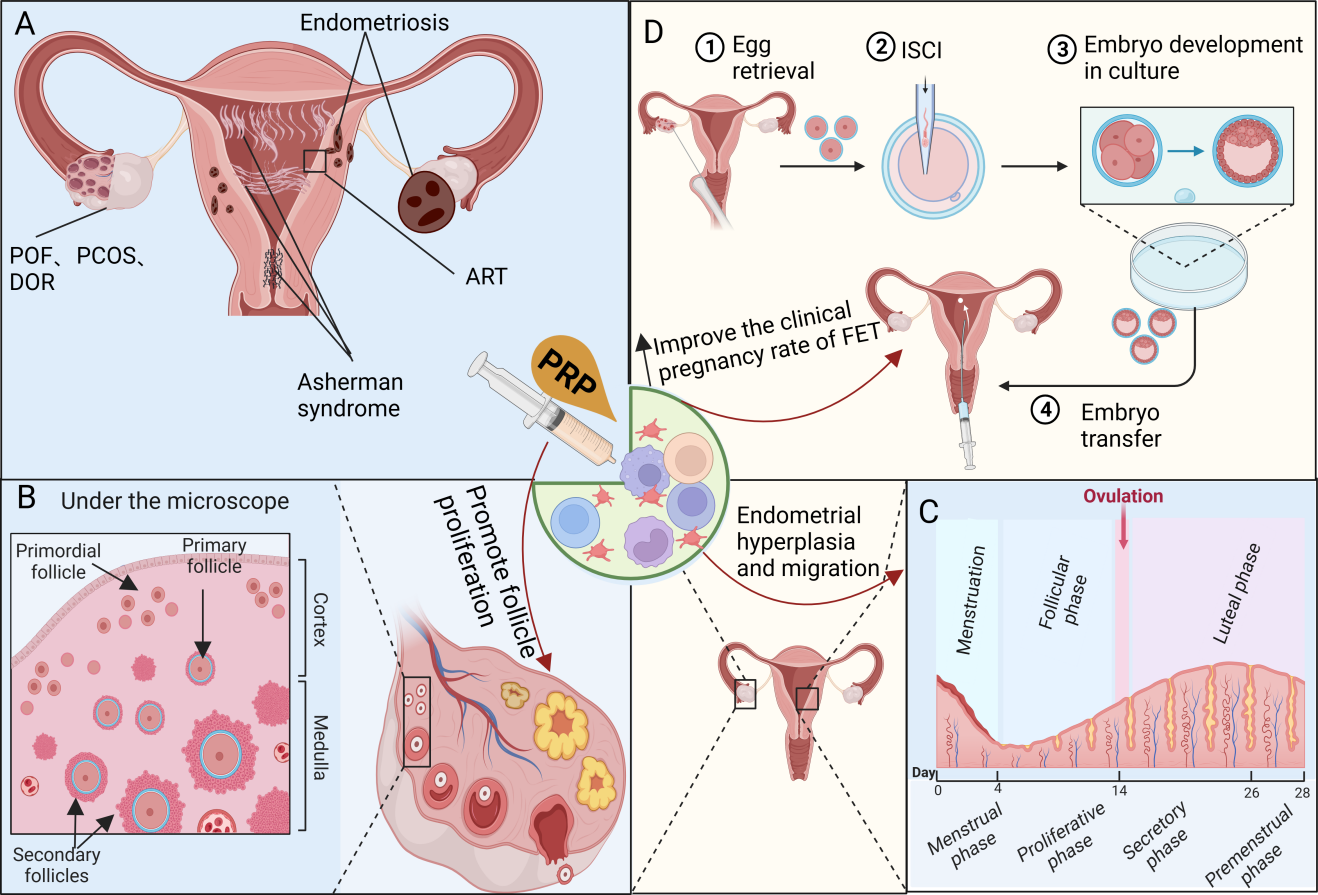
Investigating the mechanisms of platelet-rich plasma (PRP) therapy for disorders related to the female reproductive and endocrine systems. **(A)** PRP therapy is commonly used to address female reproductive and endocrine dysfunctions such as POF, PCOS, DOR, Asherman syndrome, and ART-related issues. **(B)** PRP promotes angiogenesis, follicle proliferation, and growth while inhibiting apoptosis, controlling inflammation, and cell migration, which may have a significant impact on the recovery of the ovarian niche. **(C)** PRP enhances the proliferation of uterine stromal, epithelial, and mesenchymal cells. The contribution of platelets to endometrial regeneration is possibly mediated by inflammation and chemoattraction, aiming to prevent widespread fibrosis and scarring. **(D)** PRP therapy improves the response of DOR or POI to ovulation-stimulating drugs while mitigating damage to the ovaries caused by chemotherapy. Furthermore, it promotes endometrial hypertrophy, enhances endometrial receptivity, and increases the clinical pregnancy rate of FET, particularly for RIF.

The roles and applications of PRP have been investigated in various female reproductive and endocrine disorders through animal and clinical studies, particularly related to ovarian and uterine disorders, as depicted in the graphic abstract (**Figure 3A**).

### 3.1 PRP therapy for reactivating ovarian function

Ovarian insufficiency presents a major challenge in reproductive medicine, resulting in impaired ovulation due to diminished follicular count (21). The innovative concept of intraovarian PRP infusion aims to restore ovarian function in perimenopausal women (22). Subsequent trials have been conducted in patients with poor ovarian response (POR), postmenopausal women, and those with POI who require intraovarian PRP infusion (23, 24). These trials demonstrate the positive impact of PRP on enhancing ovarian function.

Currently, PRP therapy is used to improve ovarian function in various conditions such as POF, perimenopausal ovarian adverse reactions, and menopausal women. In perimenopausal patients, intraovarian PRP injections resulted in the successful recovery of the menstrual cycle and oocytes, along with successful fertilization of mature oocytes through intracytoplasmic sperm injection following 1–3 treatment cycles (22). A randomized controlled trial demonstrated that 18 out of 30 patients with POI experienced menstrual recovery after three cycles of PRP treatment, accompanied by significant improvements in anti-Mullerian hormone (AMH), follicle-stimulating hormone (FSH), and antral follicle count (AFC) levels (23). Similarly, 13 out of 30 menopausal women responded positively to PRP treatment. In the end, 24 of the 30 perimenopausal women reported improvements in menstrual regularity, hormone levels, and AFC (24). Remarkably, a 40-year-old woman who had been experiencing premature menopause since the age of 35 opted for PRP treatment to promote ovarian tissue rejuvenation instead of oocyte donation. Six weeks after autologous PRP therapy, a notable decrease in the patient’s FSH level was observed. However, spontaneous abortion occurred in week 5 (25). In addition to improving ovarian function, a single intraovarian injection of stem cells combined with activated PRP stimulated follicle activation and development in young and old mice without adverse effects on oocytes in normal ovaries (26). A systematic review concluded that intraovarian PRP infusion effectively rejuvenated the ovaries and improved ovarian reserve parameters, leading to increased serum AMH and AFC levels and decreased serum FSH levels (23).

These studies demonstrated that intraovarian PRP infusion can restore ovarian function, reactivate follicle formation, normalize menstrual cycles, and improve hormonal status. This can be an option for some women who seek to use their gametes to achieve pregnancy or conceive naturally. Conducting well-designed randomized controlled trials is crucial to identifying the profiles of women who can benefit from the clinical application of PRP.

### 3.2 PRP therapy for PCOS

PCOS is a common endocrine disorder affecting women of reproductive age and is characterized by elevated serum androgen levels, chronic anovulation, and polycystic morphological changes in the ovaries (27).

PRP has been demonstrated to effectively alleviate PCOS pathogenesis in female rats. PRP inhibits excess androgen synthesis and ameliorates hormone dysregulation, resulting in significant reductions in FSH, luteinizing hormone (LH), testosterone, and androstenedione levels, as well as substantial increases in estrogen (E2) and progesterone synthesis. Additionally, PRP improves ovarian antioxidant status and inhibits c-Myc overexpression, leading to an increased ovulation rate (17). Fortunately, in a case study involving a woman with long-term amenorrhea due to PCOS, spontaneous ovulation cycles were restored following intraovarian PRP administration, and several aspects of hormonal imbalance were improved. Dynamic monitoring demonstrated elevated E2 levels following PRP injection, and ultrasound monitoring revealed the presence of dominant follicles and endometrial thickening (28). The potential mechanisms underlying the improvement of PCOS following PRP administration include: PRP exerts its effects on the ovaries by upregulating the expression of ERα and ERβ receptors in granulosa cells, thereby preventing follicular atresia; antioxidant chemicals are utilized to manage or reduce PCOS-induced pathogenesis (17); PRP may have local effects on follicle production through growth factors, immunomodulators, and other cytokines (4–6, 13).

PRP’s ability to regulate hormonal interactions, improve ovarian antioxidant potential, and locally enhance follicle generation can be considered a novel approach to preventing and improving PCOS pathogenesis. This approach holds promise for enhancing fertility, long-term health, and quality of life in patients with PCOS and contributes to individualized treatment and long-term management.

### 3.3 Protective effect of PRP on ovarian failure induced by chemotherapy

Ovarian damage is a significant long-term issue in children and young women undergoing chemotherapy. Alkylating agents such as cyclophosphamide (CYC) have pronounced gonadal toxicity, leading to ovarian failure in over 40% of treated women (29). Preserving the fertility of women undergoing chemotherapy is crucial. Given the gradual recognition of PRP’s role in regenerative medicine and ovarian function recovery, few studies have explored the role of PRP in preventing reproductive toxicity in patients receiving chemotherapy drugs.

PRP can enhance the ovarian reserve by protecting ovarian granulosa cells from CYC-induced damage. Moreover, PRP can stimulate and improve oocyte count and embryo development because of oocyte stimulation during IVF (30). Rats treated with CYC exhibited substantially lower serum AMH levels than those treated with CYC + PRP. Furthermore, CYC + PRP can increase the number of primary, secondary, and sinus follicles (31). The testicular structure of adult rats, in which testicular albinism was induced by oxymethoxone, showed partial improvement in vacuolation reduction and spermatogenic cell regeneration, and the sperm morphology was reasonably improved. Following two PRP treatments, histological slides demonstrated significant restoration of normal testicular structure and regeneration of spermatogenic cells; most sperm morphologies were normal (14). CYC not only impairs ovarian function but also markedly inhibits uterine function, leading to a reduction in uterine weight. Adding lyophilized equine PRP reduced CYC-induced serum nitric oxide and malondialdehyde levels. Body weight and ovarian and uterine morphometry change (32).

PRP, an autologous product abundant in various growth factors, exhibits a potent antioxidant capacity and protective effects on the reproductive system, particularly in preventing ovarian damage caused by CYC and other drugs. In addition, oocyte stimulation during IVF can improve oocyte count and embryo development (30). Therefore, studying the effects of PRP in preventing reproductive organ injury is of great clinical significance.

### 3.4 PRP therapy for intrauterine adhesion

Women of reproductive age experience periodic shedding and subsequent regeneration throughout the ovarian cycle without scarring (**Figure 3B**). The repair process requires the coordinated involvement of fibroblasts, epithelial cells, endothelial cells, adult stem/progenitor cells, as well as microenvironmental responses. These include cell proliferation, migration, differentiation, and transdifferentiation through the mesenchymal-epithelial transition (12, 33), and are associated with endometrial disease, infertility, and adverse pregnancy outcomes (34, 35). AS also known as intrauterine adhesion (IUA), is an organic disease caused by improper endometrial surgery or infection. This can lead to amenorrhea, infertility, and poor pregnancy outcomes of ART in women of childbearing age and has become one of the main uterine factors causing infertility (36). At present, the comprehensive treatment of IUA focuses on removing adhesive bands and repairing the shape of the uterine cavity and emphasizes the repair and regeneration of the damaged endometrium. However, PRP’s application in treating IUA is still preliminary, and its effectiveness as an adjunctive therapy after the hysteroscopic release of IUA remains inconclusive. This paper aimeds to review and summarize previous PRP treatments for IUA to provide evidence for the application of PRP in treating IUA.

Activated platelet α particles release cytokines and growth factors to create a suitable biological microenvironment in the endometrium, facilitating the repair of damaged tissues (9, 15). Intrauterine PRP injections after hysteroscopic adhesiolysis can improve the clinical pregnancy rate in patients with moderate to severe IUA (5).I In addition to IUA treatment with hysteroscopy and HRT, the mean endometrial thickness (EMT) in the PRP group was significantly increased (P < 0.001) (9). Activated PRP enhances different cell types associated with endometrial hypertrophy. This includes the migration of human endometrial stromal fibroblasts (eSF), endometrial mesenchymal stem cells (MSCs), bone marrow-derived MSCs, and Ishikawa endometrial adenocarcinoma cells to promote the proliferation of MSCs and mesenchymal cells. In endometrial MSCs and eSFs, activated PRP increased the expression of matrix metalloproteinases (MMPs). Simultaneously, activated PRP upregulated the transcription of inflammatory markers and chemokines compared with platelet-poor plasma (15). Platelet-derived factors are critical for endometrial progenitor cell activity, and PDGF subtypes significantly promote the proliferation, migration, and contractile force of endometrial stromal cells (ESCs) (37). This PDGF subtype may promote endometrial tissue repair by enhancing the proliferation and expansion of ESCs, stimulating ESC migration, and stimulating ESCs to shrink the collagen gel matrix (37). PRP promotes endometrial cell proliferation and migration, which forms the basis for endometrial regeneration.

Since 2015, reports have shown improvements in EMT and successful pregnancy/delivery after using PRP for assisted fertility treatment. Autologous PRP does not require culture and is known for its ease of preparation and safety. PRP treatment is believed to aid endometrial regeneration, increase uterine blood flow, and alleviate endometritis. A combined treatment of PRP with hysteroscopy or HRT can further enhance the prognosis of IUA and reduce recurrence.

### 3.5 PRP therapy for cesarean section

Wound complications after cesarean section are a common cause of morbidity in the puerperal period, particularly due to risk factors (e.g., twins, chronic systemic diseases such as diabetes, hypertension, immune deficiency, operation duration > 90 min, obesity, previous incision, corticosteroid and immunosuppressive therapy, and anemia) that impact wound healing (38). The surgical community recommends the autologous application of PRP as a new method to prevent postoperative wound infections, enhance wound healing, and alleviate pain, with emerging benefits in cesarean section applications.

Owing to its high platelet and growth factor content, PRP is a potent treatment for wound healing and is anticipated to expedite the healing process following cesarean section. The redness, edema, ecchymosis, discharge, approximation (REEDA) scores in the PRP group were markedly lower than those in the control group. The PRP group exhibited less inflammation than the control group, which was characterized by decreased redness, swelling, and secretion. At 6 months, the PRP group also experienced a substantial reduction in REEDA scores and demonstrated superior cosmetic appearance and appropriate wound closure compared with the control group. Starting on day 7, the PRP group exhibited significantly lower pain scores on the Vancouver Scar Scale (VSS) and the Visual Analog Scale (VAS). The lower VAS pain scores continued after 6 months, in contrast to the control group (39). Another study demonstrating analogous outcomes observed a more substantial decrease in REEDA scores in the PRP group than in the control group. The PRP group exhibited a notable decline in VSS scores from day 5 post-cesarean section, maintaining this trend through week 8. In addition, at the end of the follow-up period, the patients treated with PRP showed a greater decline in their VAS scores. PRP has beneficial effects on wound healing and pain reduction in high-risk patients undergoing cesarean delivery, particularly in low-resource settings (40).

PRP, characterized by a high platelet concentration, enhances adhesion and wound healing. Superphysiological platelet concentration at the wound site accelerates healing and diminishes the likelihood of postoperative wound infection. PRP is an effective treatment for wound healing in cesarean section patients, particularly those with multiple high-risk factors, and is anticipated to expedite healing owing to its enriched platelet and growth factor content.

### 3.6 Effect of PRP treatment on assisted reproductive outcomes in females

Despite significant advancements in ART, challenges, such as low ovarian response and repeated implantation failure (RIF), persist. The gradual application of PRP in ART has shown promise for enhancing ovarian and endometrial proliferation. However, more extensive studies with larger sample sizes and more comprehensive pregnancy outcome data are necessary to conclusively determine the effectiveness of PRP in ART.

#### 3.6.1 PRP therapy for decreased ovarian reserve or POI

Ovarian aging leads to a steady decline in the quantity and quality of oocyte reserves, imposing a significant constraint on spontaneous conception and ART success (41). Promising advancements in treating patients with infertility and POF using PRP have prompted reproductive physicians to further investigate the effects of PRP on women with infertility and decreased ovarian reserve (DOR) or POI (23).

Women aged > 40 with a POR who received PRP treatment exhibited a notable increase in the number of oocytes and embryos and elevated estradiol levels without significant alterations in serum FSH, LH, or AMH levels (8). A clinical study involving 311 women with POI who received PRP injections revealed that 7.4% achieved natural conception, whereas 64.8% developed antral follicles and pursued IVF. Only 27.8% of patients did not require additional treatment because of the absence of antral follicle development. Among those who underwent IVF, 82 produced embryos, of which 25 patients opted for cryopreservation for future transfer, and 57 underwent embryo transfer, resulting in 13 successful pregnancies. Additionally, 25 women treated with PRP achieved live births or continuous implantation (spontaneously or after IVF) (42). A study conducted with a broader patient age range (N = 510; age range 30–45 years) revealed that PRP treatment in women with POR (mean age 40.3 years) resulted in favorable enhancements in ovarian reserve parameters and pregnancy rates, particularly in older patients (43). Conversely, other studies reported higher overall biochemical and clinical pregnancy rates in the PRP group, with no disparity in early miscarriage or live birth rates (44). These findings suggest that intraovarian injection of autologous PRP can be an alternative experimental treatment option for women with POI.

PRP holds significance in the realm of reproductive medicine as an emerging therapy with the potential to address the persistent challenges of poor ovarian reserves and genetically linked infant issues. It is imperative to conduct high-quality randomized controlled trials to assess PRP’s efficacy in clinical pregnancy and live birth rates. Moreover, patients should be stratified, and suitable ovarian reserve markers should be identified to determine the subgroups that would benefit most from PRP.

#### 3.6.2 PRP therapy for RIF

Despite notable advancements in ART in recent years, RIF remains a prominent challenge (9). The application of PRP in women undergoing ART has been previously explored, yielding varying degrees of success. Therefore, there is a need to investigate whether the intrauterine injection of PRP enhances pregnancy outcomes in women undergoing ART (**Figures 3C, D**).

In assessing factors influencing IVF pregnancy, the endometrium is pivotal in the IVF success rate. EMT serves as an indicator of endometrial receptivity and a prognostic factor for implantation. The recommended EMT for embryo transfer is at least 7 mm or larger after the follicular phase, as the pregnancy rate for women with EMT ≤ 6 mm is less than 30%. Recent studies have focused on treating refractory thin endometria using ART to enhance implantation and live birth rates. A combination of PRP and HRT facilitated endometrial thickening in patients with a thin endometrium (≤ 7 mm). Out of the 36 patients who underwent treatment, 32 (88.9%) underwent frozen embryo transfer (FET), resulting in a clinical pregnancy rate of 15.6% with no adverse events (9). Patients with a refractory thin endometrium (< 7 mm) who had not responded to standard drug therapy after more than two cycles and were candidates for IVF cycles were given intrauterine PRP. A significant increase in EMT and enhanced endometrial pattern were observed after intrauterine PRP infusion. Continued pregnancy, chemical pregnancy, clinical pregnancy, and implantation rates also increased significantly after PRP infusion, whereas miscarriage rates decreased (18). Intrauterine infusion of PRP before embryo transfer during the FET cycle can significantly improve live birth and clinical pregnancy rates in patients with RIF. Although the implantation rate of the PRP group was higher than that of the control group, and the spontaneous abortion rate was lower than that of the control group, the difference was not statistically significant. Notably, there were no differences in pregnancy outcomes between the two groups of patients with PCOS and RIF (45). Optimal EMT is a key factor in successful implantation and pregnancy, and the treatment of refractory thin endometria during IVF is a relatively challenging issue. Autologous intrauterine PRP infusion is an alternative adjuvant therapy that enhances the EMT and echo patterns. Its upregulation in ESCs can improve endometrial receptivity, making it a novel and potentially successful treatment for thin endometria in women undergoing ART (**Figure 3C**).

Researchers have postulated that intrauterine perfusion of PRP expedites endometrial growth, stimulates the formation of endometrial spiral arteries, and augments endometrial blood flow. This enhances the nutrient supply for embryo implantation and improves endometrial receptivity, thereby increasing the clinical pregnancy rate (**Figures 3C, D**) (45). Nonetheless, additional studies with larger sample sizes and amalgamated data on pregnancy outcomes are required to establish definitive conclusions regarding the efficacy of PRP in addressing RIF.

#### 3.6.3 PRP therapy for ovarian tissue culture *in vitro*

Ovarian tissue cryopreservation is an alternative method for preserving fertility in women of reproductive age and preadolescent girls who are not at risk of hormonal stimulation or reintroduction of cancer cells, thereby preserving pre-sinus follicles. The viability and development of pre-sinus follicles are crucial for the success of the procedure and represent one of the primary limiting factors for ovarian tissue cryopreservation. Numerous studies have demonstrated the positive impact of PRP on the growth and differentiation of various cell types (13) and have advocated its use as a substitute for fetal bovine serum in cell culture (46). The growth and survival rates of the follicles in the fresh and vitrified groups were significantly higher than those in the other groups. Furthermore, adding PRP to the medium improved the survival and growth of early precaval follicles *in vitro* (47). Moreover, autologous transplantation of frozen/thawed human ovarian tissues treated with PRP has led to live births (48). Human preantral follicles were isolated from ovarian medullary tissue and cultured for 8 days. The survival rate of follicles in the human platelet lysate (HPL) group was significantly higher compared with the fetal bovine serum, human serum albumin, and umbilical cord plasma groups. Additionally, the median growth of viable follicles was significantly greater in the HPL group, although the AMH and E2 analyses did not reveal any differences between the groups. Consequently, HPL significantly enhanced the survival and growth of cultured human preantral follicles (49).

PRP more effectively supports the *in vitro* viability and growth of original human and primary follicles and represents a more favorable source of growth factors, potentially serving as a promising tool for preserving female fertility. However, the application of PRP in the field of reproduction necessitates further in-depth studies and follow-up of large samples. Additional research on the precise mechanism of PRP treatment is warranted to obtain more conclusive evidence and clarify any potential risks it may pose.

### 3.7 Study of PRP and antiplatelet treatment for endometriosis and adenomyosis

Recently, the understanding of adenomyosis and endometriosis has improved. Adenomyosis and endometriosis, experiencing periodic bleeding similar to the endometrial lining, can be considered as wounds undergoing repeated tissue damage and repair (ReTIAR) (50). Platelets quickly gather at the injury site to initiate hemostasis and tissue repair processes involving inflammation, proliferation, and remodeling (11). Elevated tissue factor immunostaining in the orthotopic and ectopic endometria of women with adenomyosis strongly suggests platelet involvement in adenoid hypertrophy (34). A Antiplatelet therapy inhibits the progression of endometriosis (51, 52).

Platelets promote hypertrophy of the *in situ* endometrium and in various endometrial locations, including the myometrium and ovaries (52). Recent studies have demonstrated the significant role of platelets in developing endometriosis (51, 53), and it has been postulated that endometriotic lesions resemble wounds undergoing ReTIAR (51). Consequently, owing to this ReTIAR, TGF-β1 derived from platelets stimulates endometriosis lesions, activating the TGF-β1/Smad3 signaling pathway, leading to EMT and fibroblast-to-myofibroblast transdifferentiation (FMT) (12). ERβ expression is upregulated in ESCs upon the aggregation and activation of platelets within endometriosis lesions (52). A Antiplatelet therapy can impede the progression of EMT, FMT, smooth muscle metaplasia (SMM), fibrosis and decrease lesion weight (54). Consistent with these findings, there was a significantly higher degree of platelet aggregation in adenomyosis and increased staining of TGF-β1 and phosphorylated Smad3. In addition, e-cadherin staining decreased, and vimentin staining increased in adenomyoepithelial cells while proliferating cell nuclear antigen, VEGF, and CD31, markers of proliferation and angiogenesis, increased. Compared with the control group, α-SMA staining was significantly increased in adenomyopathic lesions, while collagen I and lysyl oxidase staining were increased, and PR-B staining was significantly decreased. Platelets and HGFs are mainly colocalized with the stromal components of adenomyosis close to the glandular epithelium (13). These findings indicate that antiplatelet therapy is promising for treating endometriosis and adenomyosis (51, 52).

Adenomyosis and endometriosis undergo EMT, FMT, and SMM, eventually leading to fibrosis facilitated by platelet aggregation and activation (54, 55). Thus, adenomyosis, endometriosis, and platelets engage in active crosstalk to sustain lesion growth and promote lesion progression and fibrosis (55). Platelets are situated at the nexus of the pro-inflammatory and regression pathways during inflammation (56), and antiplatelet or anticoagulant strategies seem logical and advantageous.

## 4 Advancements in PRP therapy for female reproductive and endocrine dysfunction

Regardless of the specific etiology of ovarian failure, the primary cause is a deficiency in stimulating primitive follicles (21). Furthermore, impaired ovarian niche function hinders its ability to support the proliferation and differentiation of granulosa cells effectively (22, 30). Studies have demonstrated that aging, POR, and POI correlate with diminished ovarian blood flow (16, 41, 57). PRP may play a crucial role in the regeneration of the ovarian niche by promoting angiogenesis, follicle proliferation and growth, inhibiting apoptosis, controlling inflammation, and regulating cell migration. Indeed, PRP may significantly contribute to the recovery of ovarian angiogenesis, leading to notable improvements in AMH, FSH, and AFC levels (4, 7, 8, 15, 30, 31). Thus, PRP can restore ovarian function in women with diverse forms of ovarian insufficiency (**Figure 3A**). In the endometrium, PRP promotes increased proliferation of uterine stromal and mesenchymal cells, as well as migration and proliferation of human endometrial epithelial (Ishikawa) cells, eSFs, endometrial MSCs, and bone marrow-derived MSCs. The involvement of platelets in endometrial regeneration may be mediated by inflammation and chemotherapy. MMPs are mainly involved in tissue reconstruction, repair, cell migration, angiogenesis, inflammatory response, wound healing, tumor invasion, and metastasis. PRP promotes the expression of MMP1, MMP3, and MMP26 in eSFs. The inflammatory markers interleukin (IL)1A, IL1B, and IL1R2 are significantly upregulated (15). By modulating ESC function during this period, PDGF may protect the endometrium from widespread fibrosis and scarring (37). In addition to promoting endometrial hypertrophy and follicular development, PRP has other mechanisms of action that improve assisted reproductive outcomes. PRP improves the response of DOR or POI to ovulation-stimulating drugs (47) and reduces ovarian damage caused by chemotherapy drugs (31). PRP therapy promotes endometrial hypertrophy, improves endometrial receptivity, and improves the clinical pregnancy rate in FET (9).

PRP shows promise in reproductive medicine by demonstrating the potential for endometrial regeneration, menstrual cycle restoration, improved follicle development, heightened endometrial receptivity (18, 19), and enhanced clinical pregnancy and live birth rates, warranting further investigation into its mechanisms of action.

## 5 Possible clinical challenges of PRP as new agents

Advantages of clinical applications (1): Multiple growth factors have a synergistic promoting effect (11, 14, 15) (2); prolonged duration of action: PRP acts as a slow-release system, gradually releasing various growth factors, thereby maintaining a prolonged impact on target cells (3); scaffold formation: protein substances like fibrin and fiber-binding protein in PRP form fiber networks and serve as scaffolds to promote cell adhesion and prevent cell loss (7) (4); PRP is self-contained, easily obtainable, and provides a safe and inexpensive preparation scheme.

Safety and Efficacy: (1) After the isolation of autologous venous blood, there is no rejection reaction or related immune rejection issues (7) (2); the various growth factors secreted by PRP also demonstrate potential hemostatic and anti-inflammatory effects (11); (3) the mechanism of action: serum growth factors do not enter the nucleus to promote cell proliferation; instead, PRP acts on the outer cell membrane to stimulate the cell, thereby promoting proliferation and enhancing healing. Hence, the likelihood of PRP causing cells to become cancerous is exceedingly low. Notably, PRP contains multiple angiogenic stimuli that promote tumor progression, and its use of PRP in cancer patients requires further exploration with caution (10). **Table 1** shows the clinical trials of FDA-approved PRP therapy, which have been initiated for female reproductive and endocrine dysfunction; therefore, prompt conclusions are anticipated.

**Table 1:** Clinical trials for treating diseases of the female reproductive system using PRP.

Study Title	Interventions	Phases	NCT Number
PRP in ICSI	PRP	NA	NCT04354363
Outcome of PRP in ICSI Patients, a Randomized Controlled Trial	PRP	2	NCT04434495
A RCT on the Effect of PRP in ICSI Patients With RIF	PRP	2	NCT04434547
PRP in RIF	PRP	NA	NCT04085783
PRP and RIF	PRP	NA	NCT03996837
PRP for Patients With RIF	PRP | Placebo	1	NCT03379649
G-CSF and PRP in Patients With RIF	G-CSF and PRP | Control	2	NCT04411212
PRP Intrauterine Infusion in Thawed Embryo Cycles	PRP | NaCL	4	NCT03734042
Assessment of Endometrial and Sub-endometrial Vascularity Before and After PRP Infusion in Frozen Embryo Transfer Cycles	PRP	NA	NCT04247204
Effect of Autologous PRP in Uterine Wound Healing	PRP | Placebo	2	NCT03497325
PRP Gel in Wound Closure in Recurrent CS	PRP gel | NaCL	1、2	NCT02775747
PRP for Uterine Scar	PRP	NA	NCT05224726
Autologous PRP to Improve Responsiveness and Embryo Quality in Patients With POR	PRP	2	NCT05105724
Autologous PRP Intra-ovarian Infusion in Poor Responders	PRP | Placebo	2、3	NCT03937661
Ovarian PRP Injection for Follicular Activation	PRP | NaCL	NA	NCT05279560
OF Following Intraovarian Injection of PRP	PRP | PPP	NA	NCT04278313
Ovarian PRP Injection in Women With POR	PRP	NA	NCT05601193
Autologous PRP Infusion May Restore OF and May Promote Folliculogenesis in POI Patients	PRP | PFP	2、3	NCT04031456
Autologous PRP Intra Ovarian Infusion in Perimenopausal Women	PRP | PFP	2、3	NCT03951194
The Efficacy of PRP for Ovarian Rejuvenation	PRP	NA	NCT03946813
Autologous PRP Infusion to Improve Outcomes in Women With Ovarian Insufficiency: a Pilot Study	PRP	NA	NCT05385848
Ovarian Injection of PRP Vs Normal Saline in POI	PRP | NaCL	1、2	NCT04922398
Autologous PRP Intraovarian Infusion for Poor Responders	PRP	2、3	NCT05181748
PRP for Endometrial Regeneration and Repair	PRP	2	NCT02825849
Study of PRP in Women With Evidence of Diminished Ovarian Reserve	PRP	NA	NCT04275700
Injections of Autologous PRP in Women With Primary Ovarian Insufficiency	PRP	NA	NCT03542708
PRP Injection Into Ovary of Patients With POI	PRP	NA	NCT04149028
Ovarian Rejuvenation for POI and POR	PRP	NA	NCT04163640
Will Autologous PRP Able To Restore OF?	PRP | NaCL	NA	NCT04381299
4-step ASCOT in POI Women to Promote Follicular Rescue	G-CSF mobilized activated PRP	3	NCT04475744
Stem Cell Therapy and Growth Factor Ovarian in Vitro Activation	PRP+BMAC	2	NCT04009473
Ovarian Rejuvenation Using PRP & Autologous tSVF and Cell Enriched tSVF	HD PRP | tSVF + PRP | Cell Enriched tSVF + PRP	1	NCT04444245
PRP on Ovarian Reserve Parameters and ICSI Outcomes in Patients With Diminished Ovarian Reserve	PRP	3	NCT06048666
Effects of Intraovarian PRP in Women With POR and POI	PRP	NA	NCT04237909
Inovium Ovarian Rejuvenation Trials	PRP	1	NCT03178695
Ovarian PRP for Diminished Ovarian Reserve	PRP	NA	NCT05790655
Autologous Intraovarian PRP Treatment in Women With POR	PRP	NA	NCT04797377
Autologous PRP and Endometrial Thickness	PRP | Tomcat catheter	2	NCT03067623
Effects of PRP on Endometrium	PRP	NA	NCT04424160
Platelet-Rich Protein and the Endometrium	PRP	NA	NCT02973555
Hysteroscopic Injections of Autologous Endometrial Cells and PRP in Patients With T-EDM	CT | PRP | PRP after CT | PRP with EC	1	NCT05455151
Use of PRP for Improving T-EDM	PRP	NA	NCT03166345
PRP Prevents Recurrence of Intrauterine Adhesions	PRP | gel	NA	NCT03629132
PRP for Insufficient Endometrium	PRP | NaCL	NA	NCT05538338
PRP Following Hysteroscopic Adhesolysis	PRP | IUB	NA	NCT03881215
Umbilical Cord Plasma for Treating Endometrial Pathologies (T-EDM/AS/Endometria Atrophy)	hUC-PRP	2	NCT05095597
The Value of Using PRP After Hysteroscopic Lysis of Severe Intrauterine Adhesions	PRP | Intrauterine Foley’s Catheter	NA	NCT03541746
Autologous Intrauterine PRP Instillation And Endometrial Scratching for Thinned Endometrium	PRP | Endometrial scratch	2	NCT04240860
Autologous PRP for Clomiphene Citrate-induced T-EDM	CC | NaCL | PRP	2、3	NCT03770026
Autologous PRP in the Management of AS	PRP | AMG | IUB	2	NCT04308811

AMG, amniotic membrane graft; AS, Asherman Syndrome; BMAC, bone marrow aspirate concentrate; BMSCs, bone marrow-derived stem cells; CC, clomiphene citrate; CT, conservative therapy; EC, endometrial cells; G-CSF, granulocyte colony-stimulating factor; HD PRP, high-density platelet-rich plasma; IUB, intrauterine balloon; ICSI, intracytoplasmic sperm injection; NaCL, saline solution; HUC, human umbilical cord; OF, ovarian function; PFP, platelet-free plasma; POI, primary ovarian insufficiency; POR, poor ovarian response; PPP, platelet-poor plasma; tSVF, tissue-derived stromal vascular fraction. NA, Not available.

Limitations: (1) Small prospective study samples and short observation times: insufficient clinical evidence data; (2) inconsistency in PRP kits: the diversity of PRP kits leads to uncertain platelet and white blood cell concentrations, lacking uniformity in preparation method and component content; (3) offspring safety: further verification is required through high-quality studies with larger sample sizes regarding the safety for offspring (4); PRP impact on ectopic endometriosis: because PRP promotes endometrial proliferation, caution should be exercised in its use with patients with ectopic endometriosis (51, 53).

In summary, using PRP in the reproductive field necessitates comprehensive follow-up studies with larger sample sizes. Further research on the precise mechanism of PRP treatment is essential to obtain conclusive evidence. Simultaneously, it is imperative to elucidate the potential risks associated with its use.

## 6 Conclusion

**Figure 4:**
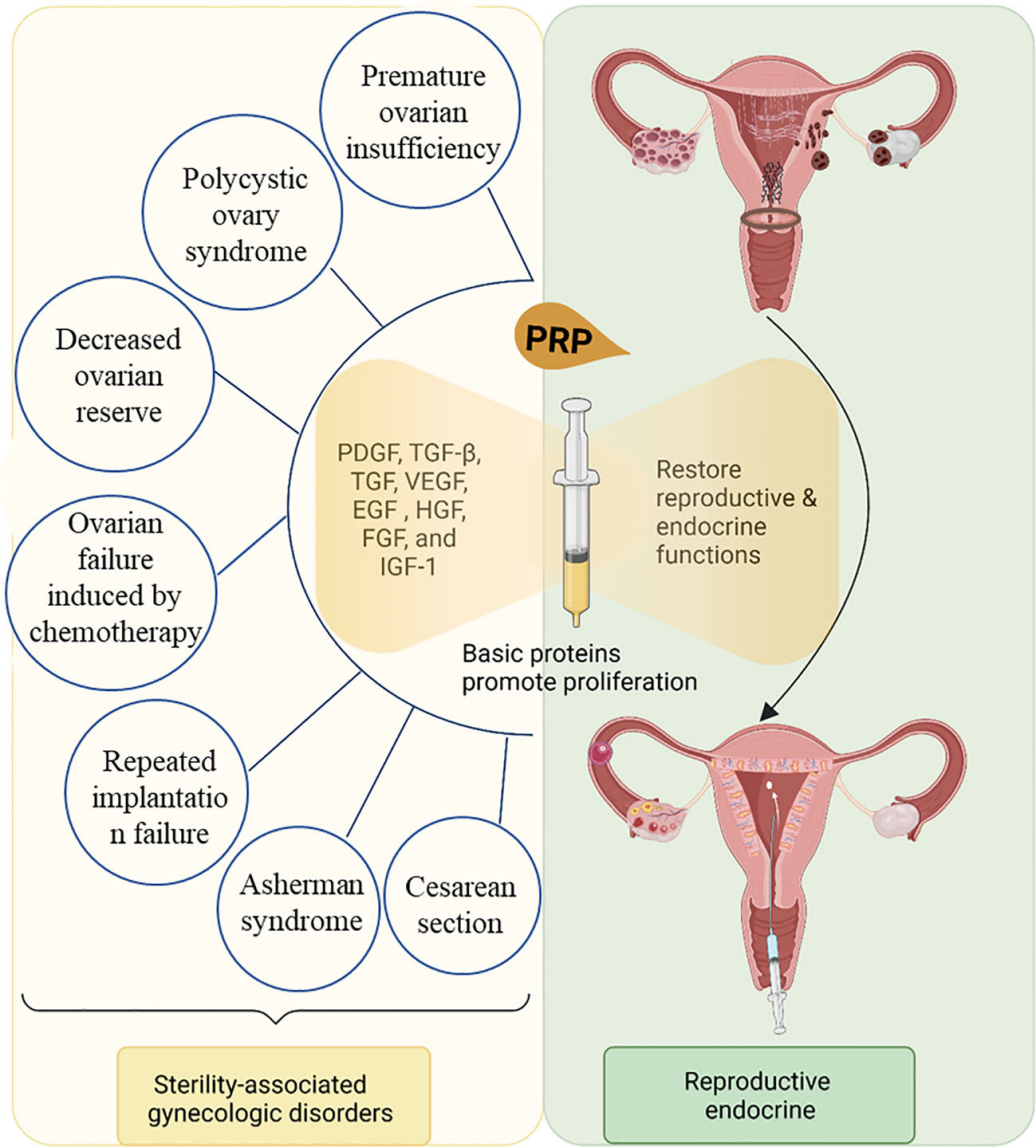
PRP is a promising therapeutic approach for female infertility and endocrine dysfunction.

Infertility poses a profound psychological burden on women and significantly affects their families and society. PRP serves as an innovative option for female infertility and endocrine disorders and presents a new treatment paradigm (**Table 2**). PRP is derived from the patient’s blood, free from side effects, and rich in growth factors and cytokines, including VEGF, PDGF, EGF, TGF-β, and other cytokines that stimulate tissue regeneration and healing. PRP augmented endometrial and vascular receptivity, exerting favorable effects on local tissue repair and the proliferation of sinus follicles. PRP presents an alternative for women facing low follicular reserve and a thin endometrium who are actively pursuing conception and represents a beneficial intervention for patients experiencing RIF (**Figure 4**).

**Table 2:** The roles of PRP in the disease of female reproductive organ.

Disease	Definition	Therapeutic roles of PRP	References
POI	The ovaries stop functioning normally before the age of 40, also known as POF	Restore OF, reactivate follicle formation, normalize MC, improve hormonal status	(22, 24–26)
PCOS	A hormonal disorder with multiple small cysts in ovaries, irregular MC, and high levels androgens	Regulate hormonal interactions Improve ovarian antioxidant Enhance follicle generation	(4–6, 13)
DOR	A condition of ovaries reduced oocytes number or diminished oocytes quality	Enhancements in ovarian reserve parameters and pregnancy rates	(32, 42, 43)
OFIC	Chemotherapy treatment induced loss of normal ovarian function	Preventing ovarian damage caused by CYC and other drugs	(14, 30–32)
RIF	Failure achieve clinical pregnancy after transfer HQE in at least three separate IVF cycles	Enhances nutrient supply for EIP Improves endometrial receptivity Increasing clinical pregnancy rate	(9, 18, 45)
AS	An organic disease caused by improper endometrial surgery or infection, lead to amenorrhea, infertility, and poor pregnancy outcomes	Aid endometrial regeneration Increase uterine blood flow Alleviate endometritis	(9, 15, 37)
CS	Surgical procedure in which baby is delivered through an incision made in the mother’s abdomen and uterus	Effective treatment for wound healing, particularly for MHRF patients	(39, 40)

PPP, Platelet Poor Plasma; POF, OF, ovarian failure; premature ovarian failure; MC, menstrual cycles; PCOS, polycystic ovary syndrome; POI, Premature Ovarian Insufficiency; DOR, decreased ovarian reserve; OFIC, ovarian failure induced by chemotherapy; CYC, cyclophosphamide; RIF, repeated implantation failure; HQE, high-quality embryos; EIP, embryo implantation; IVF, in vitro fertilization; AS, Asherman Syndrome; CS, Cesarean section; MHRF, multiple high-risk factors.

PRP has been used in clinical practice for nearly 40 years, and according to long-term clinical experience, it is safe and effective for trauma repair. Therefore, applying PRP in treating female reproductive endocrine diseases has good prospects. Currently, the clinical utilization of PRP in treating uterine adhesions, ovarian function activation, and other conditions is nascent, and its efficacy remains debatable. In particular, its impact on offspring necessitates large-scale randomized controlled trials and extensive long-term clinical observations. Furthermore, there is a lack of uniformity in the PRP application mode, timing, dosage, and frequency. Extensive clinical studies are required to determine the optimal approach for using PRP to foster endometrial hypertrophy, ovarian rejuvenation, assisted reproduction, and post-cesarean section recuperation. Therefore, a thorough examination of the mechanism by which PRP promotes ovarian and endometrial hypertrophy is required. Ultimately, PRP may introduce new therapeutic concepts and options to address female reproductive, endocrine dysfunctions and pregnancy-related diseases (threatened abortion, threatened preterm birth, hypertensive disorders during pregnancy, gestational diabetes, intrauterine growth restriction, etc.) in the near future (**Figure 4**). However, more research is needed to fully understand its benefits and establish evidence-based guidelines for its use. As research progresses, it is hoped that PRP may offer new therapeutic options for women’s reproductive health.

## Author contributions

JJL: Funding acquisition, Visualization, Writing – original draft. JL: Data curation, Project administration, Validation, Visualization, Writing – review & editing. WL: Software, Supervision, Writing – review & editing. FG: Formal analysis, Software, Writing – review & editing. XW: Conceptualization, Methodology, Writing – original draft. SZ: Conceptualization, Supervision, Writing – review & editing.
